# Impact of right ventricular pacing site on the subcutaneous ICD sensing—a step towards personalised device therapy?

**DOI:** 10.1007/s10840-022-01218-9

**Published:** 2022-04-23

**Authors:** Mohamed ElRefai, Mohamed Abouelasaad, Christina Menexi, John Morgan, Paul R. Roberts

**Affiliations:** 1 Cardiac Rhythm Management Research Department, University Hospital Southampton NHS Foundation Trust, Southampton, UK; 2 Faculty of Medicine, University of Southampton, Southampton, UK

**Keywords:** Leadless pacemakers, Cardiac implantable devices, Subcutaneous implantable cardiac defibrillators, Personalised medicine

## Abstract

**Background:**

Patients with an existing subcutaneous implantable cardiac defibrillator (S-ICD) may develop a pacing indication. When transvenous pacing is not feasible, combining an S-ICD and a leadless pacemaker (LP) can be a reasonable option. There are reports of concomitant use of both devices. However, the effect of pacing on the S-ICD sensing is not well studied. We hypothesise that pacing changes R and T-wave amplitudes, causing changes in R:T ratios as perceived by a S-ICD, increasing the risk for T wave oversensing (TWO) during paced rhythm with a subsequent risk of inappropriate shocks.

**Methods:**

This is a prospective study in patients undergoing electrophysiological studies. Participants were fitted with a Holter®, and the leads were placed to correspond to the vectors of an S-ICD. The right ventricle was paced at four positions for 10 beats each at 8 mA/2 ms. The Holter® traces were analysed, using two-way analysis of variance (ANOVA) to assess the effect of pacing on the R:T ratio.

**Results:**

Forty-seven patients (age 56.02 ± 16.02, 72% male) were enrolled (81% structurally normal heart, 15% dilated cardiomyopathy, 2% ischaemic cardiomyopathy, and 2% adult congenital heart disease). Age, sex, and aetiology had no effect on the R:T ratio. Pacing caused significant changes in the R:T ratio. There was no significant difference in the R:T ratios between the pacing sites (*p* < 0.001).

**Conclusions:**

Pacing alters the R:T ratio significantly in most patients, theoretically increasing the risk for TWO and inappropriate shocks. Tailored programming for both devices is important for concomitant use of LPs and S-ICDs.

## Condensed abstract

The concomitant use of leadless pacemakers (LPs) with subcutaneous implantable cardiac defibrillators (S-ICDs) is a tempting option when implanting transvenous leads is not desirable or feasible; however, the effect of pacing on the S-ICD sensing function was not well studied before. In our work, we illustrate the potential of oversensing in S-ICD patients who require pacing. Further work needs to be done to address the potential clinical implications of our findings. We recommend that the implant procedures as well as the devices programming be tailored for each individual patient.

## Background

### S-ICD indications and eligibility

In the absence of need for anti-tachycardia pacing (ATP), S-ICD therapy could be more beneficial compared with transvenous implantable cardiac defibrillator (TV-ICD) therapy by avoiding transvenous lead complications. However, not all the patients are eligible for S-ICD therapy. The eligibility for S-ICD is identified during a mandatory pre-implant screening process that is undertaken in all potential S-ICD recipients using guidelines by the device manufacturer.

The vectors sensed by the S-ICD strongly resemble a surface electrocardiography (ECG), and the individual ECG components can be easily visually identified. The main morphological determinant of eligibility is the relative amplitudes of the R wave and the T wave, with small R:T ratios being unacceptable. The R:T ratio is determined by the position from which an electrocardiogram is recorded as varying the axis of recording alters the amplitude of both R wave and T wave. The S-ICD senses three distinct vectors: primary, secondary, and alternate, and each has a unique R:T ratio.

For screening purposes, clinicians use surface ECG recordings as a surrogate marker of future S-ICD vectors to be able to non-invasively assess vector morphology and determine S-ICD eligibility. Patients with an ECG morphology that does not meet the screening criteria are deemed to be at such high risk of T Wave oversensing (TWO) that they are ineligible for an S-ICD. Small R:T ratio is the most common cause of screening failure [1, 2].

### S-ICD paired with a pacemaker

Almost 90% of their complications associated with cardiac implantable devices (CIEDs) are related to the presence of endovascular leads and device pocket issues, such as erosion and infection [3–5]. Occasionally, it can sometimes be difficult to implant transvenous CIEDs due to access issues such as difficult underlying anatomy or vascular occlusions. Also, in some cases implanting transvenous devices poses a high risk of infection particularly in patients with prior history of device-related infections.

Improvements in battery technology helped in the development of leadless pacemakers. Leadless pacemaker registries demonstrated high implantation success rates (99.1%), and a low rate of major complications of only 2.7% associated with leadless pacing. The overall reliability and safety profile of leadless pacing were comparable to that of traditional pacing [6, 7].

Cardiovascular diseases tend to run a progressive course, and patients with an implanted S-ICD might subsequently develop a clinical indication for pacing or vice versa when a patient with a pacemaker in situ develop a clinical need for defibrillation protection, and since S-ICDs are not suitable to provide reliable pacing, the options would be to extract the S-ICD and place a TV-ICD instead for dual function as ICD and a pacemaker or through placing a concomitant pacemaker to act independent from the S-ICD by providing bradycardia pacing therapy to the patient.

It is important to note that patients who have S-ICD implants are likely to share factors that would either preclude them from having a transvenous lead or rendering having a transvenous lead highly undesirable such as high risk of infection, difficult anatomy, difficult venous access, young age with anticipated decades of requirement for defibrillator therapy. This makes the option of extracting S-ICD and placing a TV-ICD less desirable and potentially less viable in real practice than placing a leadless pacemaker which can be an elegant approach to deliver both bradycardia pacing and defibrillation without the need for leads in the vasculature. Prospects of the modular cardiac rhythm management (mCRM) system are expected to include communicating leadless devices to provide dual chamber pacing therapy or even cardiac resynchronization therapy with the potential of coordination with a co-implanted S-ICD. For the time being, leadless pacing systems and S-ICDs act independently. There are a few reports in the literature of the use of leadless pacing with S-ICD [8, 9].

### Concerns with concomitant use of both devices

There are some concerns with the concomitant use of both devices. There is lack of data on how a shock from the S-ICD can affect the pacemaker function. In addition, pacing within the right ventricle changes the surface ECG significantly. It is possible that these changes to the ECG morphology may lead to significant changes in R:T ratio as perceived by an S-ICD. This theoretically can lead to TWO and double counting which could lead to inappropriate shocks. This is important as inappropriate shock therapies can have detrimental effects on the quality of life, psychological wellbeing, and can even result in the induction of ventricular arrhythmias [10]. Through our study, we aim to identify if there could be a benefit in tailoring a leadless pacemaker implant position in patients with concomitant S-ICDs to mitigate any potential adverse outcomes as a consequence of interaction between both devices.

## Methods

This is a prospective observational study to assess the effect of right ventricular pacing on the R:T ratio from the S-ICD perspective. The objectives of the study were, first, to determine if pacing has a significant impact on the R wave and/or T wave amplitudes and subsequently R:T ratios in the paced beats. Second, to determine if changing the pacing location has a significant impact on the R:T ratios in the paced beats. Third, to quantify the difference in the R:T ratios in the paced beats in different pacing locations. Then, subsequently, identify the pacemaker positions that would result in the most favourable R:T ratios in the paced beats. Favourable R:T ratios would impose the least risk of T-Wave oversensing by a concomitant S-ICD.

The study was performed with ethical approval from Health Research Authority (HRA)—REC (20/NW/0366)—and was also granted local research and development (RHMCAR0528) approval. All patients gave informed written consent prior to recruitment in the study. Patients’ demographics were obtained from the patients’ medical records.

**Fig. 1:**
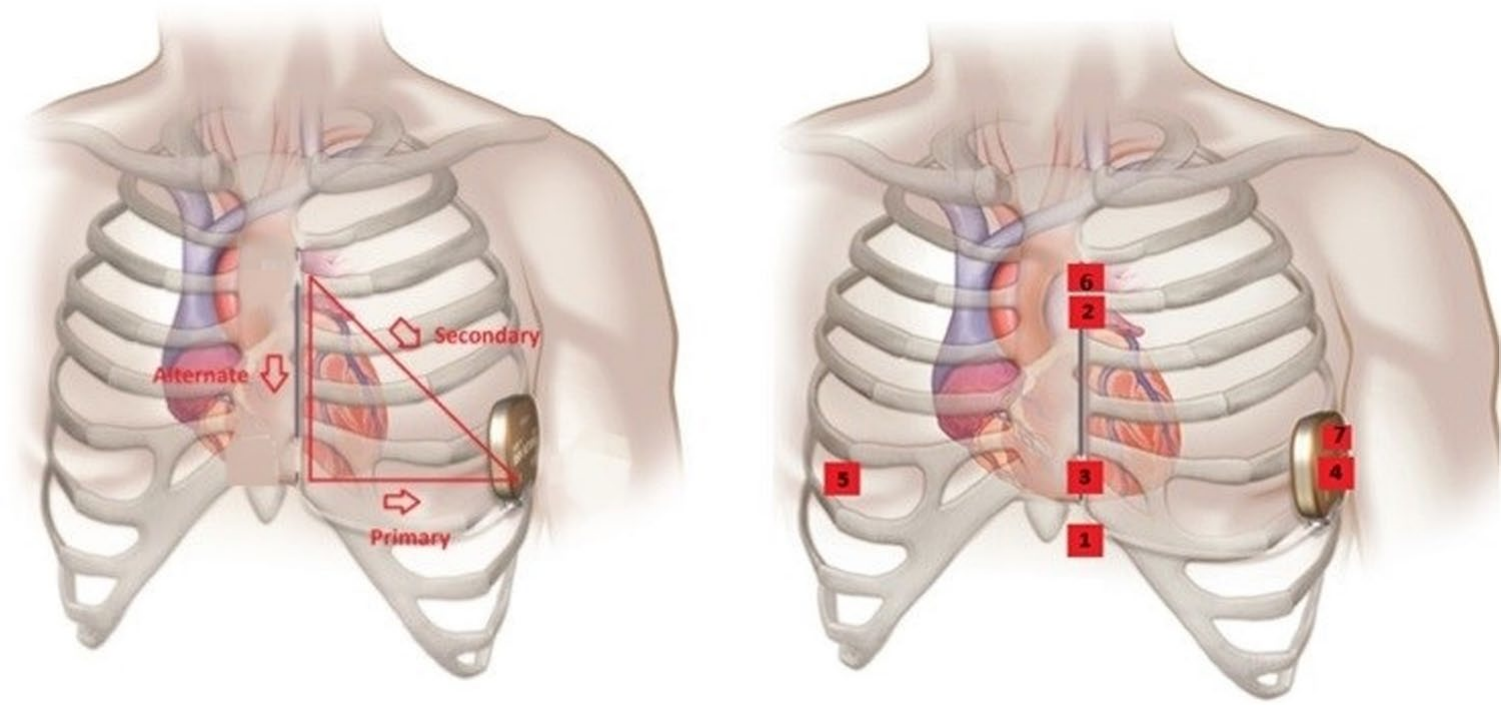
Showing the typical S-ICD vectors on the left and on the right, the Holter® surface ECG positions. 1 = 1 cm infero-lateral to the xiphisternum, 2 = 14 cm superior to position 1, 3 = 5^th^ intercostal space, parasternal position, 4 = 6^th^ intercostal space left mid axillary line, 6 = Adjacent to 2, 7 = Adjacent to 4, Holter Channel A records between points 1 and 4 = surrogate of S-ICD primary vector, Holter Channel B records between points 2 and 3 = surrogate of S-ICD alternate vector, Holter Channel C records between points 6 and 7 = surrogate of S-ICD secondary vector, 5 = 5^th^ intercostal space right mid clavicular line = neutral electrode, Image prior to annotation © Boston Scientific Corporation or its affiliates

**Fig. 2:**
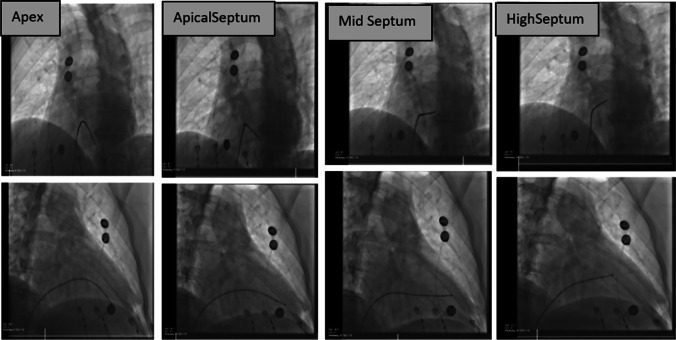
Fluoroscopic images in the left anterior oblique (top row) and right anterior oblique (bottom row) views showing the pacing catheter placed at four different sites in the right ventricle corresponding to the potential implantation sites for the leadless pacemaker

Consecutive patients who were undergoing invasive electrophysiological studies on clinical grounds were recruited. Every recruited participant was fitted with a seven-lead, three-channel Holter device prior to their clinical procedure. The leads for the Holter device were placed in a way such as the three recorded channels corresponded to the three distinct sensing vectors of an S-ICD, namely primary (from proximal electrode ring to can), alternate (from distal to proximal electrode), and secondary (from distal electrode ring to can) vectors, see Fig. 1. At the beginning or towards the end of the clinically indicated electrophysiology procedure, at the discretion of the operator, the right ventricle was paced at four different locations—true apex, apical septum, mid septum, and high septum—for 10 beats at each position at the same rate (10 beats above the resting heart rate) and using the same parameters (8 mA/2 ms) for all the paced beats. The positions of the pacing catheter were confirmed by the operator using multiple fluoroscopic views, see Fig. 2. The pacing impulses were delivered using a standard conventional pacing catheter to mimic the pacing impulses that would be delivered by a pacemaker. The four different pacing locations that were chosen for the study were based on the most common leadless pacemaker implantation sites reported in the Micra post approval registry [11].

The Holter data was downloaded, and the three channels—corresponding to the three vectors of an S-ICD—were analysed specifically for the R wave and T wave amplitudes in the non-paced beats as well as in the paced beats at the four pre-specified pacing locations using Cardio Calipers™: an on-screen ECG measurement software. A R:T ratio cutoff of 3:1 was chosen following the manufacturer guidelines for the S-ICD screening threshold [1]. R:T ratio of < 3:1 was considered unfavourable as they increase the risk of TWO.

### Statistical analysis

Data was analysed using R programme. Normality tests, histograms and boxplots were used to define parametric and non-parametric data. Parametric data was presented as mean ± SD, and non-parametric data was presented as median (IQR). Wilcoxon rank sum test was used to compare continuous non-parametric data between different groups. Kruskal–Wallis rank sum and Pearson’s chi-squared tests were used to determine the significance in the difference between different vectors in the same pacing site and in the non-pacing group. Dunn test was used for subgroup post hoc analysis with *p* value adjusted using Bonferroni method.

## Results

A total of 141 vectors were obtained from 47 recruited patients. The mean age was 56.0 ± 16.0 years, 72% male. Thirty-eight patients had a structurally normal heart, 7 patients had dilated cardiomyopathy, one patient had underlying ischemic cardiomyopathy, and one patient had adult congenital heart disease. Twenty-nine patients had underlying normal sinus rhythm, 15 had atrial fibrillation, and 3 had atrial flutter, see Table 1. No statistical significance was found when comparing the R:T ratio between different age groups, underlying rhythm, or gender. Patients with structurally normal hearts had statistically significant lower R:T ratios (2.3 (1.8, 4.0)) when compared with patients with underlying cardiomyopathy (2.7 (2.0, 4.1)) (*p* = 0.015).

**Table 1:** Patients’ demographics

	*N* = 47^*1*^
Sex	
F	13 (28%)
M	34 (72%)
Age (years)	56.02 ± 16.02
Underlying aetiology	
Adult congenital heart disease	1 (2.1%)
Dilated cardiomyopathy	7 (15%)
Ischaemic cardiomyopathy	1 (2.1%)
Structurally normal heart	38 (81%)
Underlying rhythm	
Atrial fibrillation	15 (32%)
Atrial flutter	3 (6.4%)
Normal sinus rhythm	29 (62%)

^*1*^*n* (%); mean ± SD

**Fig. 3:**
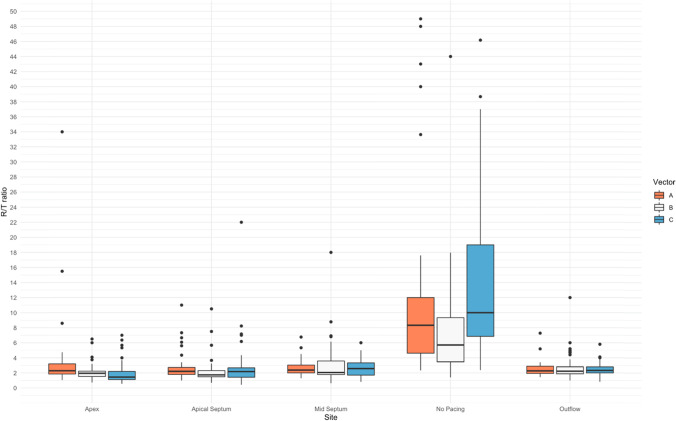
Boxplot comparing between different pacing sites and the “no pacing” group. Significant decrease was noticed in the R:T ratio when pacing in any of the selected sites. Multiple outliers were detected in all the pacing sites with nearly isoelectric T waves. Vectors A, B, and C correspond to Primary, alternate, and secondary vectors of an S-ICD, respectively

The median R:T ratio for all the vectors combined at the baseline without pacing was 7.8 (4.6,12.0), significantly higher than the median R:T ratios at different pacing sites: 2.4(1.8,3.2) pacing at the mid-septum, 2.3(1.9,2.9) at the septal outflow, 2.0(1.6,2.7) at the apical septum, and 1.9 (1.4,2.6) at the apex (*p* < 0.001). Pacing—regardless of pacing site—caused significant decrease in the R:T ratio, *p* < 0.0001, see Table 2 and Fig. 3.

**Table 2:** Comparison between the R:T ratios at different pacing sites and with no pacing

Characteristic	Pacing site					*p *value^*2*^
	Apex, *N* = 141^*1*^	Apical septum, *N* = 141^*1*^	Mid septum, *N* = 141^*1*^	No pacing, *N* = 141^*1*^	Outflow, *N* = 141^*1*^	
R:T ratio	1.9 (1.4, 2.6)	2.0 (1.6, 2.7)	2.4 (1.8, 3.2)	7.8 (4.6, 12.0)	2.3 (1.9, 2.9)	** < 0.001**
Favourable R:T ratio (> 3:1)	27 (20%)	23 (17%)	38 (27%)	124 (89%)	32 (23%)	** < 0.001**

The data in bold highlights results wit statistical significance (*p* value less than 0.05)

^*1*^Median (IQR); *n* (%)

^*2*^Kruskal-Wallis rank sum tests; Pearson’s chi-squared test

*N* = 141, number of vectors analysed in our study obtained from 47 recruited patients

Eight-nine percent of the vectors exhibited favourable (> 3:1) R:T ratios in the absence of pacing; this percentage reduced significantly with pacing: 27% with pacing at the mid-septum, 23% at the septal outflow, 20% at the apex, and 17% at the apical septum (*p* < 0.001), see Table 2.

There was a statistically significant difference in the median R:T ratios between the different pacing sites. Dunn test was used for subgroup post hoc analysis. It showed that both outflow and mid-septum pacing sites had higher R:T ratios (2.3 and 2.4 respectively) in comparison with the apical and apical septum (1.9 and 2.0), see Table 3. However, upon comparing the number of cases with favourable R:T ratio (> 3), there was no significant difference between different pacing sites, see Table 4.

**Table 3:** Post hoc subgroup analyses for the R:T ratio for different pacing sites

	Apex	Apical septum	Mid-septum
Apical septum	− 1.068602 0.8557		
Mid septum	− 3.981633 0.0002*	− 2.934529 0.0100*	
Outflow	− 3.892788 0.0003*	− 2.845028 0.0133*	0.089500 1.0000

Dunn test with Bonferroni adjustment

alpha = 0.05

Reject Ho if *p* ≤ alpha/2

**Table 4:** Comparison between the R:T ratios at different pacing sites

	Site				*p* value^*2*^
	Apex, *N* = 141^***1***^	Apical septum, *N* = 141^*1*^	Mid septum, *N* = 141^*1*^	Outflow, *N* = 141^*1*^	
R:T ratio	1.93 (1.43–2.60)	2.00 (1.58–2.67)	2.36 (1.82–3.24)	2.28 (1.94–2.85)	< 0.001
R:T ratio > 3:1	27 (20)	23 (17)	38 (27)	32 (23)	0.16

^*1*^Median (IQR); *n* (%)

^*2*^Kruskal-Wallis rank sum test; Pearson’s chi-squared test

The median R:T ratios for the primary, alternate, and secondary vectors were also assessed separately; the secondary vector had the highest R:T ratio without pacing (10 (7,19)) followed by the primary vector (8(5,12)) then the alternate vector (6(3,9)), *p* = 0.001. During pacing at the apex, the primary vector had the highest R:T ratio (2.29(1.85,3.21)), followed by the alternate vector (1.96(1.53,2.24)) then the secondary vector (1.44(1.12,2.20)), *p* < 0.001. Pacing at the apical septum resulted in a highest R:T ratio at the primary vector (2.21(1.80,2.73)), followed by the secondary vector (2.17(1.43,2.67)) then the alternate vector (1.74(1.49,2.31)), *p* = 0.07. Mid-septal pacing resulted in the highest R:T ratios at the secondary vector (2.57(1.71,3.33)), followed by the primary vector (2.37(2.00,3.04)) then the alternate vector (2.05(1.79,3.58)), *p* = 0.5. There was very little difference between the vectors in the median R:T ratios during pacing at the septal outflow: 2.27(1.93,2.89) in the primary, 2.22(1.88,2.81) in the alternate, and 2.33(2.00,2.80) in the secondary vectors, *p* = 0.9, Table 5.

**Table 5:** Comparison between the R:T ratio in the three distinct S-ICD vectors

Site	Vectors (R:T ratio)			*p*value^*2*^
	A (Primary), *N* = 47^*1*^	B (Alternate), *N* = 47^*1*^	C (Secondary), *N* = 47^*1*^	
Apex	2.29 (1.85, 3.21)	1.96 (1.53, 2.24)	1.44 (1.12, 2.20)	** < 0.001**
Apical septum	2.21 (1.80, 2.73)	1.74 (1.49, 2.31)	2.17 (1.43, 2.67)	0.07
Mid-septum	2.37 (2.00, 3.04)	2.05 (1.79, 3.58)	2.57 (1.71, 3.33)	0.5
Outflow	2.27 (1.93, 2.89)	2.22 (1.88, 2.81)	2.33 (2.00, 2.80)	0.9
No pacing	8 (5, 12)	6 (3, 9)	10 (7, 19)	**0.001**

The data in bold highlights results wit statistical significance (*p* value less than 0.05)

^*1*^Median (IQR)

^*2*^Kruskal-Wallis rank sum tests

In the absence of pacing, the secondary vector had the highest (96%) probability of exhibiting a favourable (> 3:1) R:T ratio, followed by the primary (91%), and then the alternate (78%) vectors, *p* = 0.02. Once pacing was instigated, these percentages significantly fall with little difference between the different vectors as follows; during pacing at the apex, the primary vector had the highest percentage of favourable R:T ratios (31%), followed by the secondary (17%), and the alternate (11%) vectors, *p* = 0.057. During pacing at the apical septum, the primary vector also had the highest percentage of favourable R:T ratios (20%), followed by the secondary (17%), and the alternate (13%), *p* = 0.7. During pacing at the mid-septum, the secondary and the alternate vectors were tied at 28% followed by the primary vector (26%), *p* > 0.9. At the septal outflow, the secondary and the alternate vectors were also tied (23%), followed by the primary vector (22%), *p* > 0.9, Table 6.

**Table 6:** Favourable R:T ratios (> 3:1) in the three distinct S-ICD vectors

Site	Vectors (R/T ratio > 3:1)			*p* value^*2*^
	A (Primary), *N* = 47^*1*^	B (Alternate), *N* = 47^*1*^	C (Secondary), *N* = 47^*1*^	
Apex	14 (31%)	5 (11%)	8 (17%)	0.057
Apical septum	9 (20%)	6 (13%)	8 (17%)	0.7
Mid-septum	12 (26%)	13 (28%)	13 (28%)	> 0.9
Outflow	10 (22%)	11 (23%)	11 (23%)	> 0.9
No pacing	43 (91%)	36 (78%)	45 (96%)	**0.022**

The data in bold highlights results wit statistical significance (*p* value less than 0.05)

^*1*^*n* (%)

^*2*^Pearson’s chi-squared tests

On the individual scale, all the patients recruited in the study had at least one vector which exhibited a favourable R:T ratio in the absence of pacing. While 81% of the patients had at least one vector with a favourable R:T ratio during pacing, 28% had 1 vector, 34% had 2 vectors, 19% had 3 vectors. Only 19% of the patients did not have any vector that exhibited favourable R:T ratios during pacing regardless of the pacing location, see Table 7.

**Table 7:** R:T ratios on the individual scale

ID	Primary vector R:T ratio						Alternate vector R:T ratio					Secondary vector R:T ratio					Vectors with favourable (> 3:1) R:T ratios	
	No	A	AS	MS	OF	No		A	AS	MS	OF	No	A	AS	MS	OF	Without pacing	During pacing
S01	5.7	1.9	2.0	2.5	2.6	6.2		1.5	2.7	2.3	2.1	4.9	1.3	4.4	2.7	2.1	3	1
S02	8.3	1.7	1.5	1.5	1.5	5.3		2.0	1.7	1.9	2.8	26.2	1.6	1.5	1.7	2.7	3	0
S03	7.7	1.9	1.4	2.0	2.3	10.5		4.1	2.0	2.8	1.9	8.9	2.3	2.0	2.7	2.0	3	1
S04	4.3	NA	2.3	2.0	1.6	5.4		NA	1.5	6.9	1.7	3.5	NA	1.8	3.6	2.0	3	2
S05	9.4	1.9	2.3	3.1	1.7	12.1		NA	1.9	1.9	1.8	26.2	3.7	2.4	2.6	2.3	3	2
S06	10.7	3.5	3.2	2.1	2.0	2.7		1.8	1.5	1.5	1.2	2.4	0.6	0.4	3.3	2.1	1	2
S07	33.6	2.0	1.9	2.1	2.0	NA		NA	NA	NA	NA	46.2	6.4	2.4	2.7	2.7	2	1
S08	7.1	8.6	6.1	3.8	5.2	3.9		2.7	2.4	2.6	1.5	10.7	1.1	1.3	1.0	3.9	3	2
S09	2.4	1.8	2.0	1.8	1.5	2.7		1.7	1.6	4.8	2.7	7.6	2.2	2.3	1.7	1.5	1	1
S10	3.1	3.2	2.7	2.9	7.3	2.1		2.2	2.2	2.1	2.4	2.4	2.4	7.2	2.3	3.5	1	2
S11	3.5	4.0	2.4	2.1	1.7	2.1		1.4	1.4	1.4	2.2	8.9	1.4	1.4	4.7	2.4	2	2
S12	16.5	4.5	4.4	2.8	3.0	8.8		1.7	1.8	3.7	5.0	11.1	0.6	2.2	5.0	4.0	3	3
S13	17.0	3.0	3.0	2.4	2.2	12.0		1.9	1.7	1.9	2.0	26.0	1.2	1.1	3.5	3.1	3	2
S14	40.0	3.3	2.6	2.1	2.8	5.4		1.6	1.7	8.8	1.9	8.2	0.7	1.1	2.7	2.3	3	2
S15	5.3	1.6	1.7	2.8	1.9	3.4		2.0	2.1	1.8	1.9	4.3	5.3	1.9	2.6	2.0	3	1
S16	6.2	2.6	1.9	2.4	2.2	18.0		1.3	1.4	2.4	2.4	11.2	2.0	2.7	2.7	2.1	3	0
S17	3.6	1.9	1.7	1.7	3.1	7.0		1.3	1.4	6.8	2.7	14.0	1.9	2.9	2.0	2.0	3	2
S18	10.7	1.7	1.6	2.4	2.0	3.8		3.2	3.7	2.2	2.0	11.5	3.2	3.5	2.4	1.9	3	2
S19	4.8	2.1	1.8	1.9	2.9	7.2		1.0	3.3	3.8	3.2	12.1	0.9	1.9	2.5	3.2	3	2
S20	11.3	2.6	6.7	6.8	3.1	6.4		2.0	2.9	2.3	2.2	18.0	1.8	2.0	3.9	2.0	3	2
S21	6.2	1.7	1.8	1.8	2.8	7.4		1.3	1.2	1.6	1.8	4.7	0.8	0.6	1.5	2.3	3	0
S22	43.0	2.3	2.1	2.0	3.1	1.5		6.5	1.6	1.9	1.9	8.8	1.0	3.2	2.4	2.5	2	3
S23	9.2	2.9	2.7	3.2	3.2	5.0		1.4	1.5	4.0	2.8	11.8	5.7	2.6	4.8	2.9	3	3
S24	10.0	2.3	2.2	2.3	2.2	14.0		2.6	2.1	2.2	4.8	16.3	1.7	2.4	2.1	5.8	3	2
S25	5.0	3.3	2.9	2.8	3.2	2.0		6.0	3.0	4.0	4.7	24.5	3.3	2.6	2.9	3.6	2	3
S26	10.5	3.1	5.6	3.6	3.2	13.0		2.1	2.8	2.0	6.0	7.0	2.1	7.0	4.8	4.1	3	3
S27	12.0	2.5	2.2	2.6	1.9	1.9		1.7	1.5	1.8	1.6	9.5	1.5	1.3	1.4	2.0	2	0
S28	49.0	4.8	1.4	5.3	2.6	7.0		2.0	1.8	1.9	3.8	22.5	1.7	1.6	1.5	3.6	3	3
S29	3.0	1.9	1.9	2.0	2.8	3.9		0.7	0.8	1.0	1.0	6.8	0.7	2.3	1.2	0.9	3	0
S30	17.6	1.8	2.8	4.2	3.4	12.8		2.0	1.6	2.0	2.3	6.9	1.4	8.2	1.3	0.8	3	2
S31	2.4	2.3	2.3	3.7	2.4	3.8		3.0	10.5	3.3	2.6	9.2	4.0	6.2	4.3	2.5	2	3
S32	48.0	15.5	11.0	4.0	2.3	10.7		2.0	1.7	1.8	1.6	23.0	1.1	1.0	1.0	2.2	3	1
S33	11.3	1.8	2.3	2.4	2.5	9.5		2.3	5.7	1.8	2.1	5.2	1.0	2.4	2.6	2.4	3	1
S34	9.5	2.8	2.4	2.8	2.7	5.4		1.3	1.5	6.1	2.7	6.9	1.3	1.8	3.4	2.6	3	2
S35	2.3	1.4	1.4	1.6	1.4	7.8		1.0	0.7	0.7	1.0	13.5	2.9	2.2	2.2	1.7	2	0
S36	5.3	2.8	2.4	2.0	1.7	6.8		2.1	1.9	1.8	2.1	38.7	1.2	1.6	3.5	2.3	3	1
S37	16.0	1.2	1.0	2.4	2.8	2.7		1.6	1.3	5.5	5.2	11.0	1.4	1.1	4.5	2.1	2	2
S38	7.2	2.0	1.9	1.8	1.9	12.7		1.5	1.3	0.6	4.4	9.2	1.3	0.8	1.9	2.1	3	1
S39	14.0	34.0	7.3	4.5	2.6	8.3		2.0	1.7	18.0	3.3	4.8	1.4	0.8	6.0	3.8	3	3
S40	7.2	3.2	3.4	3.2	3.1	44.0		3.8	3.2	4.3	12.0	37.0	7.0	22.0	2.9	2.6	3	3
S41	12.0	3.9	2.2	4.3	2.2	6.0		2.2	2.3	2.7	2.0	20.0	0.8	2.8	1.7	2.3	3	1
S42	14.7	2.6	2.0	2.3	2.1	4.5		2.4	7.5	1.8	2.3	6.0	2.0	2.8	2.6	2.0	3	1
S43	3.8	NA	NA	NA	NA	3.0		1.7	1.6	1.6	2.2	10.0	1.7	2.3	1.5	1.8	3	0
S44	3.5	1.4	1.3	1.3	1.6	1.4		1.4	1.3	1.3	1.6	3.8	1.4	1.7	1.7	1.7	2	0
S45	9.6	1.9	2.7	2.4	1.6	3.3		2.3	2.2	1.5	1.3	4.3	0.9	0.9	0.8	3.5	3	1
S46	4.4	1.1	2.0	2.1	2.1	12.0		2.0	1.9	2.3	3.8	25.0	1.2	2.4	2.5	1.9	3	1
S47	4.0	2.0	1.7	2.0	2.0	4.9		1.5	1.5	1.5	2.1	24.0	1.3	2.1	2.6	2.4	3	0

*ID study ID, No no pacing, A apical pacing, AS apical septum pacing, MS mid-septum pacing, OF septal outflow pacing*

## Discussion

The effect of pacing on the sensing process of the S-ICD is not well studied, and the effect of pacing specifically on the R:T ratio as perceived by the S-ICD is not currently known. Changes in the R wave and T wave amplitudes can lead to changes in the R:T ratio which is vital to the sensing mechanism of the S-ICD. In this study, we were particularly interested in the changes associated with R:T ratios because of pacing as well as changing the location of pacing. R:T ratio was chosen specifically as the parameter to be analysed because of the crucial role of the R:T ratio in the sensing mechanism of the S-ICD and its subsequent determination of S-ICD eligibility and TWO events. Even though the patient would have previously passed the S-ICD screening, it is possible that QRS double counting or T wave oversensing could occur during paced rhythm. In this case there is a potential risk of inappropriate shock due to oversensing of paced rhythm. This may be particularly relevant in certain pacing circumstances. For example, a patient programmed with hysteresis may see a rapid jump from 45 to 90 bpm. If there were TWO, this may produce a perceived sudden onset of a heart rate of 180 bpm which may be within the detection zone of the programming of the S-ICD. Aside from few sporadic cases published as case reports [8, 9], we are not aware of any studies that looked specifically into the cohort of patients who have leadless pacemakers in situ who would be deemed eligible for an S-ICD if they develop an indication for one.

## Pacemaker position effect on ECG signals

Current practices favour implanting the leadless pacemaker into the trabeculated septal wall and avoid the right ventricular free wall or the right ventricular apex; this is to minimise the perceived risk of perforation. Leadless pacemaker registries have demonstrated wide variation in the implantation sites of leadless pacemakers [11]. Further data analysis demonstrated no variation in the short and intermediate term performance of the leadless pacemakers regardless of the implantation site [12, 13]. As such, there is no preference in the leadless pacemaker deployment location as long as the thinner right ventricular free wall is avoided.

While pacing is known to be associated with ECG morphological changes, the effect of changing the pacing location on certain morphological aspects of the ECG is not well studied. The specific locations for pacing that were chosen for our study were based on the leadless pacemakers’ implant locations reported in the registries.^11^ Our aim was to identify the most favourable location that would either lead to a minimal effect on the ECG morphology and R:T ratios or even if associated with significant changes, still maintain a favourable R:T ratio from a S-ICD perspective.

## Data analysis

**Fig. 4:**
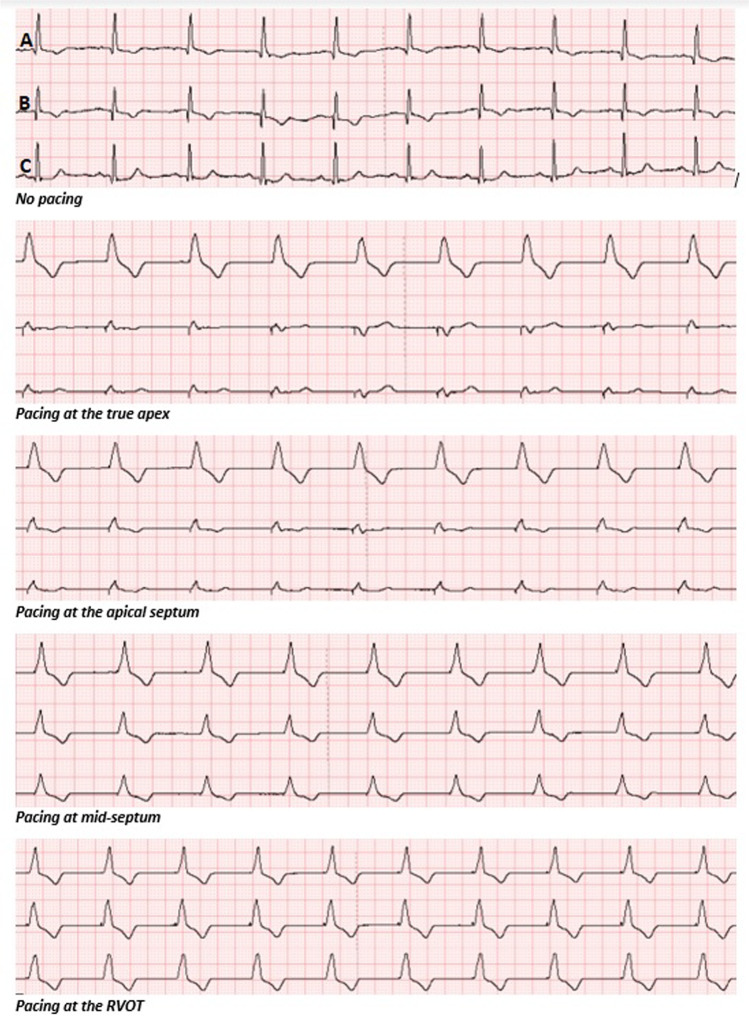
An example of the effect of pacing as well as changing the pacing site on the morphology of the Holter traces corresponding to the S-ICD vectors. A, B, and C correspond to primary, alternate, and secondary vectors respectively

We have demonstrated through our study that first pacing caused significant changes in the ECG morphology, particularly in the R:T ratios when looked at from an S-ICD perspective. Second, pacing site also had an impact on the morphological changes associated with pacing, and R:T ratios perceived by all the S-ICD vectors changed with changing the pacing site, see Fig. 4. Pacing, regardless of the pacing site, in general had a detrimental effect on the R:T ratio perceived by the S-ICD vectors. This is clearly demonstrated as the median R:T ratios in all three vectors and across all the pacing sites have fallen below 3:1 once pacing was instigated. Most (89%) of the recorded vectors had a favourable R:T ratio (> 3:1) prior to pacing in our cohort of recruited patients. This high percentage drastically falls once pacing is initiated; the average percentage of favourable R:T ratios in all vectors at all pacing locations once pacing was instigated was only 21%.

However, and despite the detrimental effect of pacing on the R:T ratio, favourable R:T ratios (> 3:1) were recorded during pacing in all 3 vectors and at all the pacing locations, albeit significantly less prevalent than in the “no pacing” traces. In fact, most (81%) of the patients recruited in the study had at least one vector that exhibited favourable R:T ratio during pacing at at least one pacing location. This means that even if the percentage of the favourable R:T ratios during pacing is overall significantly less when looking at all vectors, the remainder favourable vectors are distributed in a way that allows 81% of the patients in our cohort to have at least one favourable/suitable vector which is enough to pass the screening. However, none of the vectors, and none of the four different pacing locations were consistently favourable (or statistically better) from the R:T ratio perspective.

When it comes to implanting a leadless pacemaker into a patient with an existing S-ICD, there is no “one size fits all”. The choice of implantation location of the leadless pacemaker as well as the choice of vector programming for the S-ICD must be tailored to each individual patient. Even in the absence of a favourable R:T ratio across all vectors and pacing locations, implanting a leadless pacemaker into a patient with an S-ICD while minimising the risk of inadvertent interaction is theoretically possible in most patients.

Furthermore, careful individualised programming of both devices is paramount. For example, the upper limit of the pacing rate of a pacemaker as well as the therapy threshold heart rate of an S-ICD should be both set—if possible—in a way that even if the pacing rate is inappropriately doubly counted, it still would not exceed the therapy rate threshold for the concomitant S-ICD. This ought to reduce the risk of inappropriate shocks due to TWO because of pacing.

## Limitations

Our study had several limitations. First, the relatively lower number of recruited patients might have hindered us from identifying a statistically significant difference in the R:T ratio between different pacing sites. Second, none of the patients recruited for the study had either or were candidates for an S-ICD or pacemaker therapy, although the same principles should still apply for them. Third, the presence of both devices (S-ICDs and leadless pacemakers) was mimicked using a Holter device with its leads placed to mimic S-ICD vectors, and a standard pacing catheter was used as a surrogate of a leadless pacemaker. This could be relevant, particularly due to the different pacing parameters between the pacing catheter (2 ms in our study) versus that of the leadless pacemaker (0.24 ms). Consequently, the paced QRS from the catheter could be potentially different from the paced QRS from the leadless pacemaker. However, exclusively recruiting patients with S-ICDs who develop a pacing indication for our study would appear to be an unjustified time-consuming process and likely to yield a lower number of recruited patients to our study even at a tertiary referral centre of our calibre specialised in implanting both S-ICDS and leadless pacemakers. In addition, most of the patients in our study had underlying structurally normal hearts, and while in real life, there are a lot of patients with S-ICDs with apparently structurally normal hearts, such as patients with underlying channelopathies, a lot of S-ICD patients have underlying cardiomyopathies of various etiologies which can affect our results, for example, the voltage and duration of a paced QRS are not the same in healthy tissue vs scar tissue. There was a statistical significance in the R:T ratio between both groups in our study. However, larger studies involving wider cohort of patients with various underlying etiologies are needed to consolidate our findings. R:T ratio of 3:1 that was used as the threshold of eligibility for our study is based on the screening threshold cutoff of the S-ICD, based on manufacturer recommendations for screening allowing for a safety margin. There is no evidence that lower R:T ratios would inevitably lead to adverse clinical events. In real life, R:T ratio might need to fall way below the proposed ratio of 3:1 for TWO to occur; however, this needs to be studied further. At last, in our study, the effect of pacing only on the R:T ratio was assessed and not other parameters such as QRS duration and QT interval, which could also potentially affect the S-ICD eligibility. However, as previously mentioned, the R:T ratio parameter was chosen for our study as a simple, easily measured parameter based on the integral role of the R:T ratio in the S-ICD sensing mechanism and determinability of S-ICD eligibility. In addition, the cutoffs at which other parameters such as QRS or QT durations would fail the S-ICD screening are not known. Further work is needed before the proposed personalised approach towards device therapy be applied in clinical practice.

## Conclusions

Extravascular and leadless devices represent the future. They have consistently demonstrated reliable performance and high safety profile avoiding the complications associated with the traditional transvenous lead-reliant devices. It is inevitable that we will see more and more patients in whom we will be tempted to utilise concomitant leadless pacemakers and S-ICDs to cover for their pacing and defibrillator protection indications rather than implanting the traditional TV-ICDs. We have demonstrated through our study that pacing, regardless of the pacing site, in general had a detrimental effect on the R:T ratio perceived by the S-ICD vectors, significantly lowering the percentages of favourable R:T ratios once pacing is instigated. However, our study also demonstrated that, at least theoretically, it is feasible in most patients to concomitantly utilise both devices if we adopt a personalised devices therapy approach. Implantation procedure for the devices as well as the devices programming needs to be tailored for every individual patient. Further work needs to be done before this can be translated into clinical practice.
